# Medical Scribe Impact on Provider Efficiency in Outpatient Radiation Oncology Clinics Before and During the COVID-19 Pandemic

**DOI:** 10.1089/tmr.2021.0035

**Published:** 2022-01-07

**Authors:** Max Devine, Elyn Wang, Rie von Eyben, Hilary P. Bagshaw

**Affiliations:** Department of Radiation Oncology, Stanford University, Stanford, California, USA.

**Keywords:** scribe, COVID-19, radiation oncology, documentation, telemedicine

## Abstract

**Purpose/Objectives:** Medical documentation has become increasingly challenging for providers, particularly with changes to telemedicine visit formats during the ongoing COVID-19 pandemic. Medical scribes may help mitigate this burden. Our objective was to determine how scribes affect provider efficiency during the COVID-19 pandemic.

**Materials/Methods:** Providers completed a survey in February 2020 (S1, prepandemic) and 1 year into the COVID-19 pandemic in February 2021 (S2, during pandemic). S1 evaluated perceived impact of scribes on clerical work, medical documentation, and efficiency during office visits using the Likert scale. S2 also addressed scribe use during telemedicine visits. Provider time spent on documentation with or without a scribe was evaluated using a five-level ordinal scale. Provider response was assessed using descriptive frequency statistics. Fisher's exact test was used to compare categorical variables. Analysis was performed using SAS version 9.4 (SAS Institute, Inc., Cary, NC). All tests were two sided with an alpha level of 0.05.

**Results:** Fifty-eight providers responded to the surveys: 36 (62%) for S1 and 22 (38%) for S2. Scribe use decreased perceived clerical work and facilitated chart review, and recording of physical examination findings, note documentation, and improved efficiency, both before and during the pandemic (*p* = 0.5, *p* = 0.7, *p* = 0.8, *p* = 0.8, *p* = 0.9, respectively). Scribe use significantly decreased time to complete documentation prepandemic (*p* = 0.002) and during the pandemic for both in-person (*p* ≤ 0.0001) and telemedicine visits (*p* = 0.0004). More providers took >60 min to complete medical documentation without the use of a scribe prepandemic (72% vs. 30% with a scribe, *p* = 0.006) and during the pandemic, after both in-person (40% vs. 0% with a scribe, *p* = 0.002) and telemedicine visits (35% vs. 0% with a scribe, *p* = 0.002).

**Conclusions:** Scribe use decreases provider time spent on medical documentation and improves overall efficiency before and during the COVID-19 pandemic for both in-person and telemedicine visits. Integration of scribes into radiation oncology in-person and telemedicine clinics may improve provider satisfaction by reducing burden of documentation.

## Introduction

The advent of the electronic health record (EHR) has brought many advantages including enhanced patient care and safety,^1^ but has also led to an increased documentation burden on physicians, and contributed to provider inefficiency, frustration, and burnout.^2,3^ During the ongoing COVID-19 pandemic, the required immediate transition to telemedicine office visits resulted in further reliance on the EHR as a means of direct patient communication in addition to documentation. It is imperative to identify ways to reduce the EHR burden on providers and optimize its utilization, especially in the current telemedicine climate, allowing clinics to run efficiently and support both provider and patient satisfaction.

Medical scribes have been increasingly utilized during inpatient and outpatient encounters as a solution to reducing the documentation burden on physicians. At our institution, medical scribes are also called patient flow coordinators. They are qualified individuals with a high school diploma or general educational development equivalent. Their training includes an introduction to the EHR and an introduction to the clinical specialty they will be working in. Their role includes accompanying a supervising physician to patient visits, reviewing the health care record with the physician, documenting the history and physical examination in the EHR, and reviewing the documentation with the physician.

Nationally, medical scribes contribute to decreased provider burnout^4–7^ and increased EHR safety,^8^ without a negative impact on patient experience.^4–6,9–11^ The benefit of medical scribes has been demonstrated across specialties including dermatology,^4,12^ family medicine/primary care,^5,6^ rheumatology and endocrinology,^11^ oncology,^7^ pediatric gastroenterology,^9^ and emergency medicine,^13–16^ however, only in in-person visits and not in telemedicine visits.

We wanted to understand the impact of medical scribes on provider workflow efficiency in radiation oncology clinics. We were specifically interested in this impact during the ongoing COVID-19 pandemic with the increased use of telemedicine clinic visits in comparison with prepandemic controls.

## Materials/Methods

In February 2020, a voluntary anonymous online survey (S1) was sent to faculty (*n* = 22), residents (*n* = 17), advanced practice providers (APPs, *n* = 3), and nurse coordinators (NCs, *n* = 13) in the department of radiation oncology regarding the utilization of the department's two medical scribes, who assist one to two faculty members per day on a rotating basis, depending on clinical need. The initial survey (Supplementary Data S1 and S2) consisted of questions referencing perceived scribe utility using a Likert scale in areas such as workload, documentation, quality of work, efficiency, and charting. A five-level ordinal scale was used to capture perceived time to review and sign notes with and without a scribe. Free text response was also allowed for additional comments.

A year later, ∼1 year into the COVID-19 pandemic, a second voluntary anonymous online survey (S2) was sent to the same providers although there were two more faculty members (*n* = 24) and one more NC (*n* = 14) in the department at this time (Supplementary Data S1 and S2). S2 consisted of questions identical to S1 and the same scribes were still working in the department. In addition, there were questions referencing the use of scribes specifically in telemedicine and in in-person clinic visits. Owing to the California Shelter-In-Place order in response to the COVID-19 pandemic,^17^ scribes began working from home. Thus, survey questions were added to evaluate clinic efficiency when scribes prepared notes ahead of time, even if they were unable to participate in the clinic visits themselves.

Provider responses for both surveys were assessed using descriptive frequency statistics. Fisher's exact test was used to compare categorical variables. Analysis was performed using SAS version 9.4 (SAS Institute, Inc., Cary, NC). All tests were two sided with an alpha level of 0.05. This was an institutional review board exempt study.

## Results

### Respondents

Thirty-six providers responded to S1. Respondents consisted of 10 faculty members (28%), 15 residents (42%), 9 NCs (25%), and 2 APPs (6%) (Table 1). Twenty-two providers responded to S2, composed of 13 faculty members (57%), 6 residents (26%), 3 NCs (13%), and 1 APP (4%) (Table 1). Response rates for each group were 45.5% (S1) and 54.2% (S2) of the faculty, 88.2% (S1) and 35.3% (S2) of residents, 66.7% (S1) and 33.3% (S2) of APPs, and 69.2% (S1) and 21.4% (S2) of NCs. Since respondents were anonymous, we were not able to directly pair any responses between S1 and S2.

**Table 1.** Respondent Demographics for Each Survey

	Survey 1 Prepandemic (*n*)	Survey 2 During pandemic (*n*)
Faculty	27.8% (10)	56.5% (13)
Resident	41.7% (15)	26.1% (6)
Nurse coordinator	25.0% (9)	13.0% (3)
APP	5.6% (2)	4.4% (1)
Total responses	36	23

Each department affiliation works with the scribes in some capacity and voluntarily completed the survey. Percentage reflects portion of responses for each individual survey.

APP, advanced practice provider.

### Scribe utility

Fisher's exact test was used to compare the six identical questions in each survey. These addressed scribe use improving efficiency, decreasing providers' daily clerical work, identifying and accurately documenting medical and historical details, accurately recording physical examination findings, noticing obvious physical examination findings, and assisting with review of charts. The majority of providers strongly agreed that scribes improved efficiency, decreased clerical work, accurately documented medical history, physical examination findings, independently identified examination abnormalities, and helped with chart review. There were no differences in responses to these questions between S1 and S2 (*p* = 0.9298, *p* = 0.5105, *p* = 0.7908, *p* = 0.3881, *p* = 0.8257, *p* = 0.7084, respectively).

### Time assistance

**FIG. 1.**
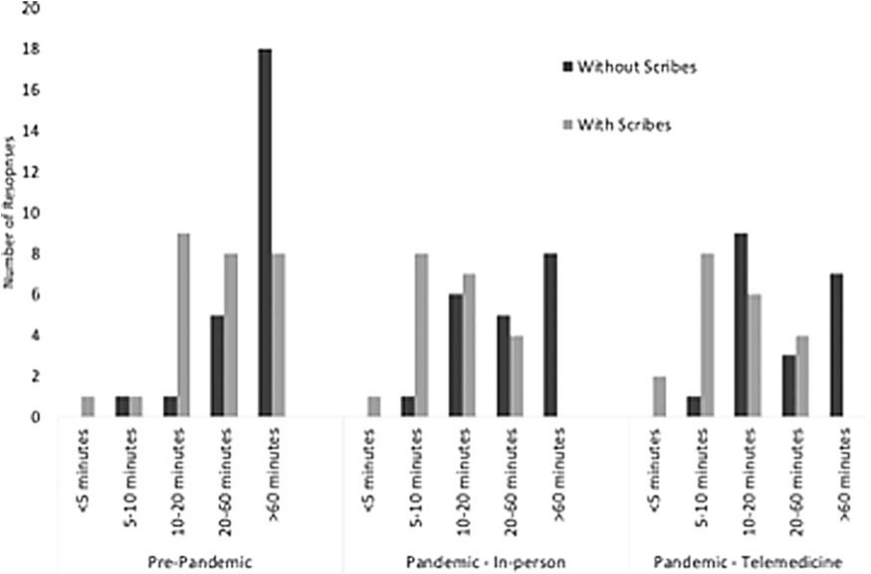
Responses to survey question: How long does it take to review and sign notes at the end of the day? Perceived time it takes providers to complete documentation at the end of a clinic day prepandemic and during the COVID-19 pandemic for both in-person and telemedicine visits.

**FIG. 2.**
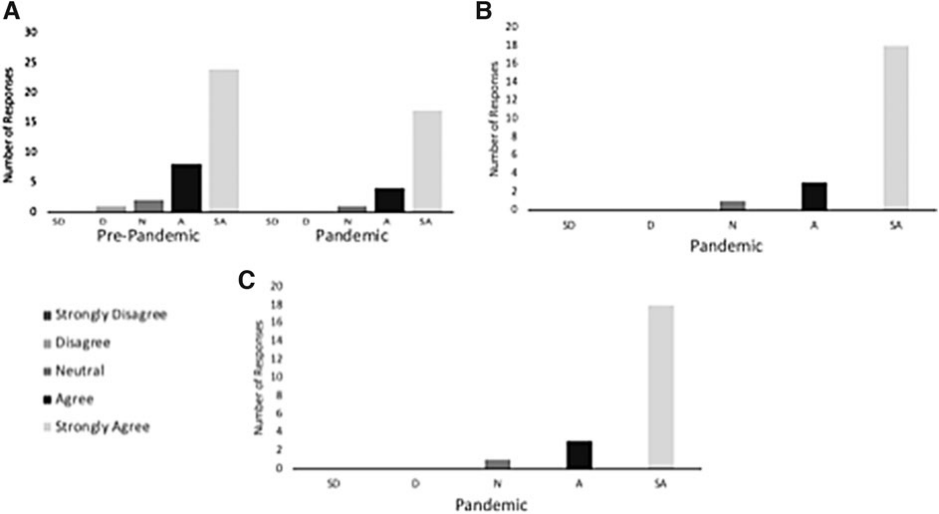
Responses to survey statement: **(A)** my scribe helps me to be more efficient (responses from both surveys). **(B)** My scribe helps me during telemedicine visits just as much as during in-person visits. **(C)** Having my notes prepared ahead of time by scribes significantly improves my efficiency during clinic, even if my scribe cannot participate in the visit. Perceived scribe utility for efficiency (both surveys), telemedicine vs. traditional visits, and note preparation.

Scribe use significantly decreased perceived time to complete documentation prepandemic (*p* = 0.002) and during the pandemic for both in-person (*p* ≤ 0.0001) and telemedicine visits (*p* = 0.0004). More providers took >60 min to complete medical documentation without the use of a scribe prepandemic (72% vs. 30% with a scribe, *p* = 0.006) and during the pandemic, after both in-person (40% vs. 0% with a scribe, *p* = 0.002) and telemedicine visits (35% vs. 0% with a scribe, *p* = 0.002) (Fig. 1). Even with increased telemedicine visits during the pandemic, 17 (77%) providers strongly agreed that scribe use decreased their daily clerical work and improved efficiency and 18 (82%) strongly agreed scribes were just as helpful during telemedicine visits as during in-person visits (Fig. 2).

### Comments

Providers were given the opportunity to provide any additional comments on the department scribe program. The overall consensus of S1 comments was that scribes played a huge role in improving efficiency and were very beneficial to the department. Even though they are not directly supported by scribes, department NCs still acknowledged how helpful they were to both the faculty and residents (*n* = 1, “Our providers find the clinic [preparation] really valuable and they are able to promptly attend other… needs if be.”) and patients (*n* = 1, “Though the [scribe] does not directly support me, I believe the position is extremely beneficial to our patient care allowing the [physician] to have meaningful discussions with the patients without having to document while in the room.”).

Faculty members also praised scribes for how much their help decreased provider time spent on writing notes (*n* = 4, “They tremendously reduce the time spent on paraphrasing other providers' notes during follow up preparation as well as the time spent on transcribing the clinical encounter.”) and increased the time they could focus on patient care (*n* = 3, “Their dedicated work significantly frees up time for treatment planning and other patient care tasks.”). Some also commented on the need to expand the program due to the help they provide (*n* = 2, “The scribe program is wonderful. We are in need of expanding it significantly to have more meaningful impact in the department.”). Resident comments were very in-depth and covered many aspects of scribe work.

Many residents commented about the lack of time to focus on other responsibilities without a scribe (*n* = 7, “Residents currently spend a large proportion of time prepping and documenting clinic visits… This adds up to many hours a week which is taken away from…activities…such as studying, research, and patient care.”) and how the presence of a scribe allows them to become better physicians (*n* = 4, “when we have a scribe it graduates us to being able to think through cases and learn.”).

All providers responding to S2 were faculty members, and the comments were overwhelmingly positive (*n* = 8, “The [scribes] are essential and greatly increases the efficiency of the faculty.”). Some recognized the importance they brought to the residency program (*n* = 4, “it has also helped with resident satisfaction and expanding the scribe program would also continue to show the residents that we support their education.”) and to patient care (*n* = 3, “keeps me on time and improves patient satisfaction.”).

There were some limitations identified by respondents to working with medical scribes. Owing to a lack of standardized training, the documentation can vary due to background and experience. Even still, these survey results show that providers still appreciate having medical scribes.

## Discussion

Radiation oncologists are more efficient when working with medical scribes. This is true prepandemic and during the pandemic, including telemedicine visits. In addition, providers report improved satisfaction and well-being, a necessary component of decreased burnout.

The improved efficiency with the use of medical scribes in radiation oncology clinics shown here is similar to those reported in many other medical specialty clinics^4–7,9,10,12–16^ but uniquely also shows they also improve efficiency in telemedicine visits. The onset of the COVID-19 pandemic led to the rapid adoption and implementation of telemedicine, forcing scribes to work remotely and adapt to a new workflow.

One hybrid in-person model recommends scribes be in a separate room from the physician and patient during visits (whether telemedicine or in-person), scribing by listening through a cell phone and thus lowering the risk of aerosol generation.^18^ A second model describes expansion of scribes' roles beyond scribing during the pandemic and the need to ensure proper training for such a format.^19^

The principle change we implemented was asking scribes to prepare notes ahead of the visit, which was overwhelmingly beneficial and providers still reported decreased time to complete documentation regardless of visit type. Importantly, patients are not only comfortable with remote scribing, one study showed that the use of face-mounted technology such as Google Glass led to patients perceiving their physicians communicating better and being more attentive.^20^ Despite limited information regarding scribe use during the pandemic, these data continue to suggest scribes are a beneficial addition to the clinical team no matter the environment.

We acknowledge limitations to this study. Owing to the voluntary nature of this survey, responses from providers could be skewed to those who had stronger feelings toward scribe usage. In addition, we were not able to receive as many responses to S2 compared with S1, and we were not able to compare responses by respondent across S1 and S2. Although the medical scribes were constant during this time, due to normal turnover in the department there could have been providers who left or joined the department between S1 and S2.

We found that medical scribe use increased provider efficiency and decreased documentation burden on providers both prepandemic and during the pandemic despite the increase in telemedicine visits. This study was performed in a radiation oncology department, but scribe utility has been shown in many other specialties as well.^4–7,9–16^

## Conclusions

Medical scribes are of great use to radiation oncology providers, improving efficiency for in-person and telemedicine clinic visits alike. With the COVID-19 pandemic altering health care settings, it is important to adapt quickly. Now more than ever, the addition or expansion of medical scribe programs could greatly improve provider satisfaction.

## Supplementary Material

Supplemental data

## Abbreviations Used

APP: advanced practice provider
EHR: electronic health care record
NC: nurse coordinator
