# Supplementary material for: Medical Scribe Impact on Provider Efficiency in Outpatient Radiation Oncology Clinics Before and During the COVID-19 Pandemic
Source: Telemed Rep. 2022 Jan 7;3(1):1–6. doi: 10.1089/tmr.2021.0035 (PMC8989091; doi:10.1089/tmr.2021.0035)
Supplement: Supplemental data [file Supp_Data.pdf]

Survey 1 (Pre-Pandemic)

Default Question Block

Please select your affiliation within the department.

- Faculty
- Resident
- Nurse Coordinator
- APP
- Other

Please indicate if you agree or disagree with the following statements. (PFC= Patient Flow Coordinator/Scribe)

	Strongly Disagree	Disagree	Neutral	Agree	Strongly agree
My PFC decreases the amount of clerical work I have to do each day.					
My PFC listens to each patient and is able to identify and accurately document pertinent medical and historical details.					
My PFC accurately records physical exam findings and assessments as I dictate them.					
My PFC notices obvious physical exam findings (e.g. nasal cannula in place, ambulates with walker) and flags them for my review.					
My PFC assists with my review of the chart, confirming details regarding history, physical examination findings, and treatment plans.					
My PFC generally completes charts within the timeframe of each appointment.					

	Strongly Disagree	Disagree	Neutral	Agree	Strongly agree
I trust the quality of the work my PFC performs.	○	○	○	○	○
My PFC helps me to be more efficient.	○	○	○	○	○
I am generally able to stay on schedule.	○	○	○	○	○
My PFC assists me in entered pended orders in the encounter so I can easily sign off prior to closing the encounter	○	○	○	○	○
My PFC assists me in entering patient instructions and the return to clinic/line care/infusion section of the chart navigator during the patient visit	○	○	○	○	○

Please indicate how long it takes to preform each task.

	Less than 5 minutes	5-10 minutes	10-20 minutes	20 - 60 minutes	More than 60 minutes
How long does it currently take you to review and sign your notes at the end of the day?	○	○	○	○	○
Before integrating a PFC into your clinical team, how long on average did it take you to review, attest and sign your notes at the end of each clinic session?	○	○	○	○	○

Please enter any additional comments regarding the scribe program in Radiation Oncology.

Survey 2 (During Pandemic)

Default Question Block

Please select your affiliation within the department.

- ○ Faculty
- ○ Resident
- ○ Nurse Coordinator
- ○ APP
- ○ Other ______

Please answer the questions below thinking about utilization of scribes/PFCs since the start of the COVID-19 pandemic, with telemedicine being used much more frequently.

	Strongly Disagree	Disagree	Neutral	Agree	Strongly Agree
Compared to before the pandemic, I currently use a PFC in clinic MORE than I used to.	○	○	○	○	○
My PFC helps me during telemedicine visits JUST AS MUCH as during in person visits.	○	○	○	○	○
Having my notes prepared ahead of time by PFCs significantly improves my efficiency during clinic, even if my PFC cannot participate in the visit.	○	○	○	○	○
My PFC helps me to be more efficient.	○	○	○	○	○

	Strongly Disagree	Disagree	Neutral	Agree	Strongly Agree
My PFC decreases the amount of clerical work I have to do each day.	○	○	○	○	○
My PFC listens to each patient and is able to identify and accurately document pertinent medical and historical details during telemedicine visits.	○	○	○	○	○
My PFC accurately records physical exam findings and assessments.	○	○	○	○	○
My PFC is able to help identify pertinent physical exam findings via telemedicine and flag them for my review.	○	○	○	○	○
My PFC assists with my review of the chart, confirming details regarding history, physical examination findings, and treatment plans.	○	○	○	○	○

Please indicate how long it takes to review and sign your notes at the end of the day in these different scenarios.

	Less than 5 minutes	5-10 minutes	10-20 minutes	20 - 60 minutes	More than 60 minutes
After in-person clinic visits	○	○	○	○	○
After in-person clinic visits having utilized a PFC	○	○	○	○	○
After telemedicine visits	○	○	○	○	○

	Less than 5 minutes	5-10 minutes	10-20 minutes	20 - 60 minutes	More than 60 minutes
After telemedicine visits having utilized a PFC	○	○	○	○	○

Please enter any additional comments regarding the scribe program in Radiation Oncology.
